# Comparative Evaluation of Combined Navigated Laser Photocoagulation and Intravitreal Ranibizumab in the Treatment of Diabetic Macular Edema

**DOI:** 10.1371/journal.pone.0113981

**Published:** 2014-12-26

**Authors:** Raffael Liegl, Julian Langer, Florian Seidensticker, Lukas Reznicek, Christos Haritoglou, Michael W. Ulbig, Aljoscha S. Neubauer, Anselm Kampik, Marcus Kernt

**Affiliations:** Department of Ophthalmology, Ludwig-Maximilians-University, Munich, Germany; Massachusetts Eye & Ear Infirmary, Harvard Medical School, United States of America

## Abstract

**Objective:**

To evaluate if a standardized combination therapy regimen, utilizing 3 monthly ranibizumab injections followed by navigated laser photocoagulation, reduces the number of total ranibizumab injections required for treatment of diabetic macular edema (DME).

**Research Design and Methods:**

A 12-month, prospective comparison of 66 patients with center-involving DME: 34 patients with combination therapy were compared to 32 patients treated with ranibizumab monotherapy. All patients initially received 3 monthly ranibizumab injections (loading phase) and additional injections pro re nata (PRN). Combination therapy patients additionally received navigated laser photocoagulation after the loading phase. Main outcome measures were mean number of injections after the loading phase and change in BCVA from baseline to month 12.

**Results:**

Navigated laser combination therapy and ranibizumab monotherapy similarly improved mean BCVA letter score (+8.41 vs. +6.31 letters, p = 0.258). In the combination group significantly less injections were required after the 3 injection loading phase (0.88±1.23 vs. 3.88±2.32, p< = 0.001). By month 12, 84% of patients in the monotherapy group had required additional ranibizumab injections as compared to 35% in the combination group (p< = 0.001).

**Conclusions:**

Navigated laser combination therapy demonstrated significant visual gains in most patients. Retreatment rate and number of injections were significantly lower compared to ranibizumab monotherapy and compared to the results of conventional laser combination therapy previously reported in pivotal anti-VEGF studies.

## Introduction

The development of antibody-derived inhibitors of vascular endothelial growth factor (VEGF), such as ranibizumab (Rbz), have dramatically changed the management of DME and progressively replaced Macular Laser Therapy (MLT) as a first-line treatment option.

Major randomized controlled trials have demonstrated that intravitreal anti-VEGF injections not only help to maintain visual acuity in patients suffering from fovea-involving DME, but can also improve vision significantly (by an average of at least six letters in the first year) [1]–[5].

The same trials have also documented that frequent intravitreal injections, on the order of seven to twelve in the first year and slightly fewer in subsequent years, were needed to accomplish and maintain these results [4], [6].

The significant treatment burden placed on patients, doctors, healthcare providers and payers, as well as, reports of inferior results with inadvertent under-treatment in everyday clinical settings, highlight the need for a treatment paradigm providing optimal visual outcomes with fewer injections [7], [8].

While conventional MLT, applied as monotherapy, demonstrated a stabilizing effect on vision at a low treatment frequency in the ETDRS and subsequent studies, so far no clear benefit has been demonstrated when added to Anti-VEGF, either with respect to enhanced visual acuity gains or reduced injection burden [4], [6], [9], [10].

A new computer-guided technology for navigated MLT, developed to overcome some of the limitations of manual, slit-lamp based laser application, has recently become available (Navilas Laser System, OD-OS GmbH, Teltow, Germany) [11]–[15].

Using digital planning and image-guidance, navigated laser therapy has demonstrated a significantly higher accuracy in laser spot application, with the potential to reduce the retreatment rate compared to conventional laser monotherapy [11]–[15].

We hypothesized, that the potential for earlier disease stabilization with navigated MLT could also translate into earlier stabilization of Anti-VEGF visual gains and therefore reduced Anti-VEGF retreatment rate and overall injection burden.

To evaluate this hypothesis, we developed a standardized treatment regimen based on the *pro re nata* (PRN) scheme of the European approval for ranibizumab and a navigated MLT application after the first three monthly injections.

## Study Population and Methods

### Study Design

This was a 12-month, prospective comparison of 66 patients with center-involving DME conducted at the Department of Ophthalmology, Ludwig-Maximilians-University, Munich, Germany. Patients either received a combination treatment consisting of ranibizumab injections plus navigated MLT or ranibizumab monotherapy as two co-existing standard treatments. Physicians that had not undergone training with navigated MLT performed Rbz monotherapy, while trained physicians performed combination therapy, leading to a quasi-random assignment of patients to their respective cohort.

The study was conducted in accordance with the Declaration of Helsinki. Approval was obtained from the institutional review board and written informed consent provided by each patient.

### Participants

Consecutive patients were enrolled in 2011 and 2012 from the outpatient clinic of the Department of Ophthalmology, Ludwig-Maximilians-University, Munich. Key eligibility criteria for all participants were: female or male with a minimum age of 18 years and a diagnosed diabetes mellitus Type I or II with clinically significant DME according to the criteria of the ETDRS [16]. Further criteria included (1) best-corrected visual acuity (BCVA) of at least 10 letters on the ETDRS chart, (2) central retinal thickening (CRT) of at least 400 µm (with foveal involvement); measured by spectral domain OCT [(SD-OCT) Spectralis OCT, Heidelberg Engineering GmbH, Heidelberg, Germany] and, based on clinical examination made by two experienced retina specialists, (3) no ischemic maculopathy seen in fluorescein angiography, (4) no severe proliferative diabetic retinopathy (PDR) or macular edema due to other underlying retinal vascular disease, vitreomacular traction or epiretinal membranes, (5) no previous anti-VEGF, MLT or other major ocular surgeries within the last 4 months, (6) no pre-existing ocular conditions that would preclude improvement in visual acuity despite reduction of the edema, (7) no pregnant or lactating subjects and (8) no subjects currently enrolled in other clinical trials.

### Standardized Treatment Regimen

All patients underwent a baseline examination including best-corrected visual acuity, slit-lamp examination, dilated fundoscopy, OCT-imaging and measurement and fluorescein angiography. We assessed BCVA at every study visit using ETDRS charts at a starting distance of 4 meters. OCT imaging was performed on spectral domain OCT with eye-tracking and rescan support in follow-up measurements (Spectralis OCT, Heidelberg Engineering GmbH, Heidelberg, Germany).

After eligibility was confirmed, all participants then received intravitreal injection therapy (IVT) with 0.5 mg ranibizumab (Rbz) closely following the European label, which details a *pro re nata* (PRN) protocol based on the RESTORE study with or without adjunct MLT. It must be noted, that this treatment paradigm differs in this regard from the U.S. label for 0.3 mg ranibizumab in DME.

As outlined above, navigated MLT was applied only to cohort 2, while cohort 1 received Rbz monotherapy:

Cohort 1 – Ranibizumab Monotherapy: Patients were initially treated with three anti-VEGF injections in one-month intervals. After the “loading phase” injections were delivered *pro re nata* (PRN).
Cohort 2 – Navigated Laser Combination Therapy: Three initial anti-VEGF injections were given in one-month intervals with a Navilas navigated MLT delivered one month after the third injection if CRT had decreased to 445 µm or below. Otherwise one more injection was given and navigated MLT applied four weeks after. Further anti-VEGF injections were delivered *pro re nata* (PRN) as described below.

According to the PRN scheme, injection therapy was paused after the loading phase in patients who demonstrated stable BCVA and CRT on two consecutive examinations, or if the BCVA reached 85 ETDRS letters or the DME was completely resolved (CRT<300 µm). Patients were followed monthly with BCVA and CRT obtained at each visit and intravitreal injections were resumed if a reduction in BCVA of more than 5 letters compared to baseline BCVA or an increase of CRT of at least 20% was observed. Retreatment was continued until BCVA and CRT were again stable for at least two consecutive visits.

### Intravitreal injections

Patients received intravitreal injections of ranibizumab as delineated above. Injections were performed according to a standard procedure: topical antibiotics were used both pre and post injection under aseptic operating theatre conditions. After draping a 30-gauge needle was inserted into the vitreous cavity through pars plana and 0.5 mg/mL RBZ were injected. The cannula was then withdrawn and a sterile cotton tip was placed on the injection site.

### Navigated MLT

Navigated MLT procedures were performed with the scanning slit laser photocoagulator, Navilas Laser System (OD-OS GmbH, Teltow, Germany), which was CE-marked and approved by the US Food and Drug Administration in 2009. Its principal operation has been described elsewhere [13]. In brief, it combines imaging, laser application planning, and treatment in a computer-based device. It fundamentally differs from other laser devices by using a scanning slit-based principle to acquire and display high-resolution images on a touch screen monitor.

Navigated MLT procedures in this study were digitally planned on Color and FA images acquired by the instrument and/or imported OCT thickness maps by placing single spots and grid patterns according to ETDRS guidelines. The spot size was typically set to 100 µm and applied with a pulse duration of 100 ms. Based on color snap images taken during treatment, laser power was individually adjusted to values around 100 mW to achieve a pale grayish, barely visible laser burn. The treatment was administered using the plan overlay and laser-beam prepositioning features of the device.

In cases were navigated MLT and anti-VEGF injections were given on the same day, we always delivered navigated MLT first and anti-VEGF injections at minimum two hours afterwards.

### Statistical Methods

All data were collected in a MS-Excel 2010 spreadsheet (Microsoft Corporation, Redmond, WA) and analyzed using the Statistical Package for Social Sciences version 21.0 for Windows (IBM, New York, USA).

## Results

**Figure 1:**
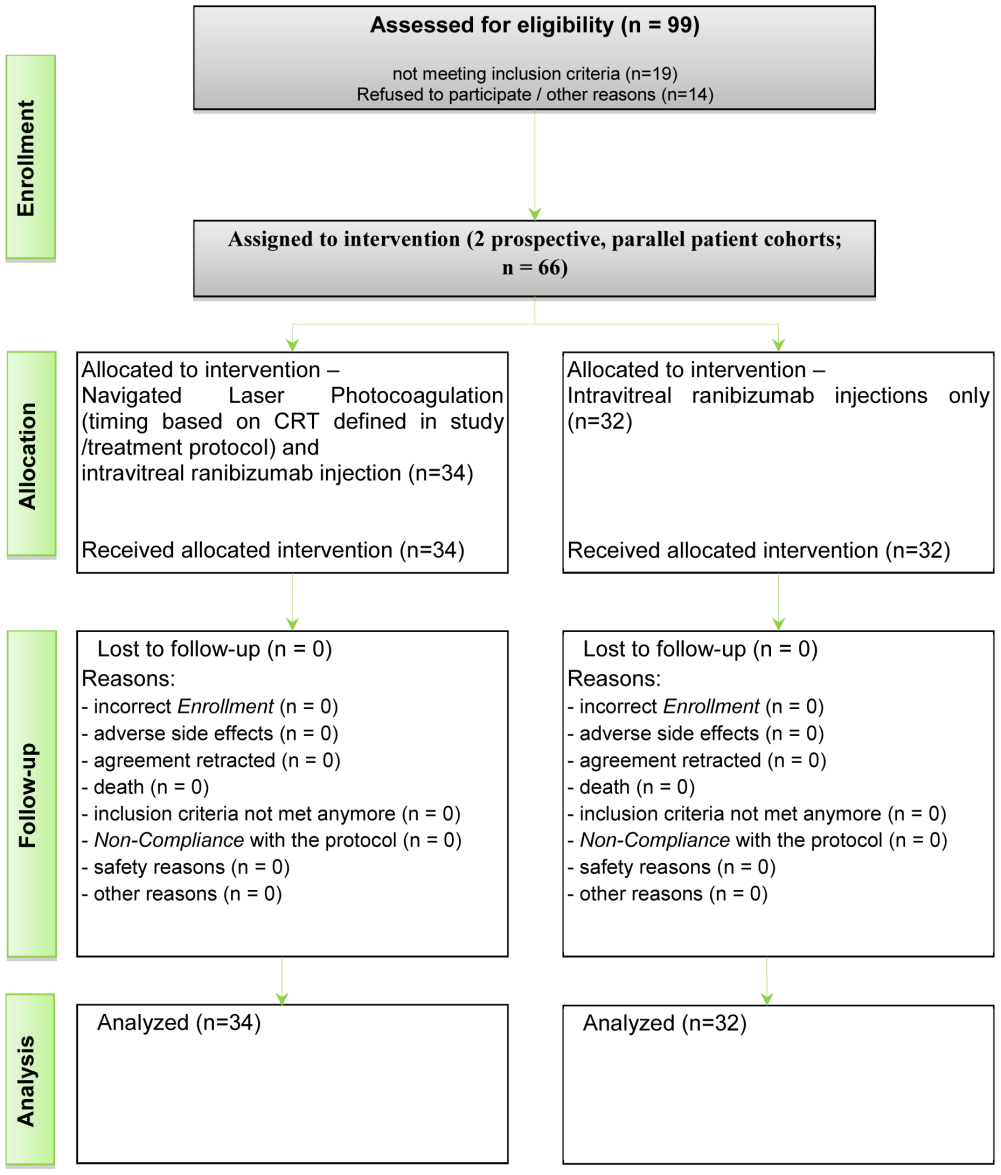
Flow chart of the study. Enrollment, Assignment and Follow-up of the patients that were included in this prospective comparison of combined navigated macula laser therapy and mono anti-VEGF therapy.

A total of 66 patients were included into this prospective comparison: 34 patients received ranibizumab/navigated laser combination therapy and 32 patients received ranibizumab monotherapy. A total of 99 patients were initially screened for this study of which 33 dropped out as they either did not meet all the inclusion criteria or refused to take part in this study. From the 66 patients that were included, none dropped out at a later stage over the study period or completely missed a follow-up. All data gathered at follow-ups were within a ten day time frame of the protocol requirements and therefore included in our study results. (Fig. 1)

Baseline characteristics, which are similar in both cohorts, are summarized in Table 1. Mean baseline BCVA was 30.8±12.6 and 24.6±14.4 letters for combination and monotherapy cohorts, respectively (p = 0.065). Mean baseline CRT values were 441±162 and 444±117, respectively (p = 0.928).

**Table 1:** Baseline Characteristics of the Sample.

	Navilas + Ranibizumab n = 34	Ranibizumab Monotherapy n = 32	Test for difference (P-Value)
Mean age ± SD, years	64.9±11.6	68.2±11.3	p = 0.255
Gender (% (n) female)	47% (16)	40% (13)	p = 0.605
Mean BCVA ± SD, ETDRS letter score	30.8±12.6	24.6±14.4	p = 0.065
Mean CRT ± SD, µm	441±162	444±117	p = 0.928

SD, standard deviation; CRT, central retinal thickness; BCVA, best corrected visual acuity; ETDRS, Early Treatment Diabetic Retinopathy Study.

### Visual Acuity and CRT development

**Figure 2:**
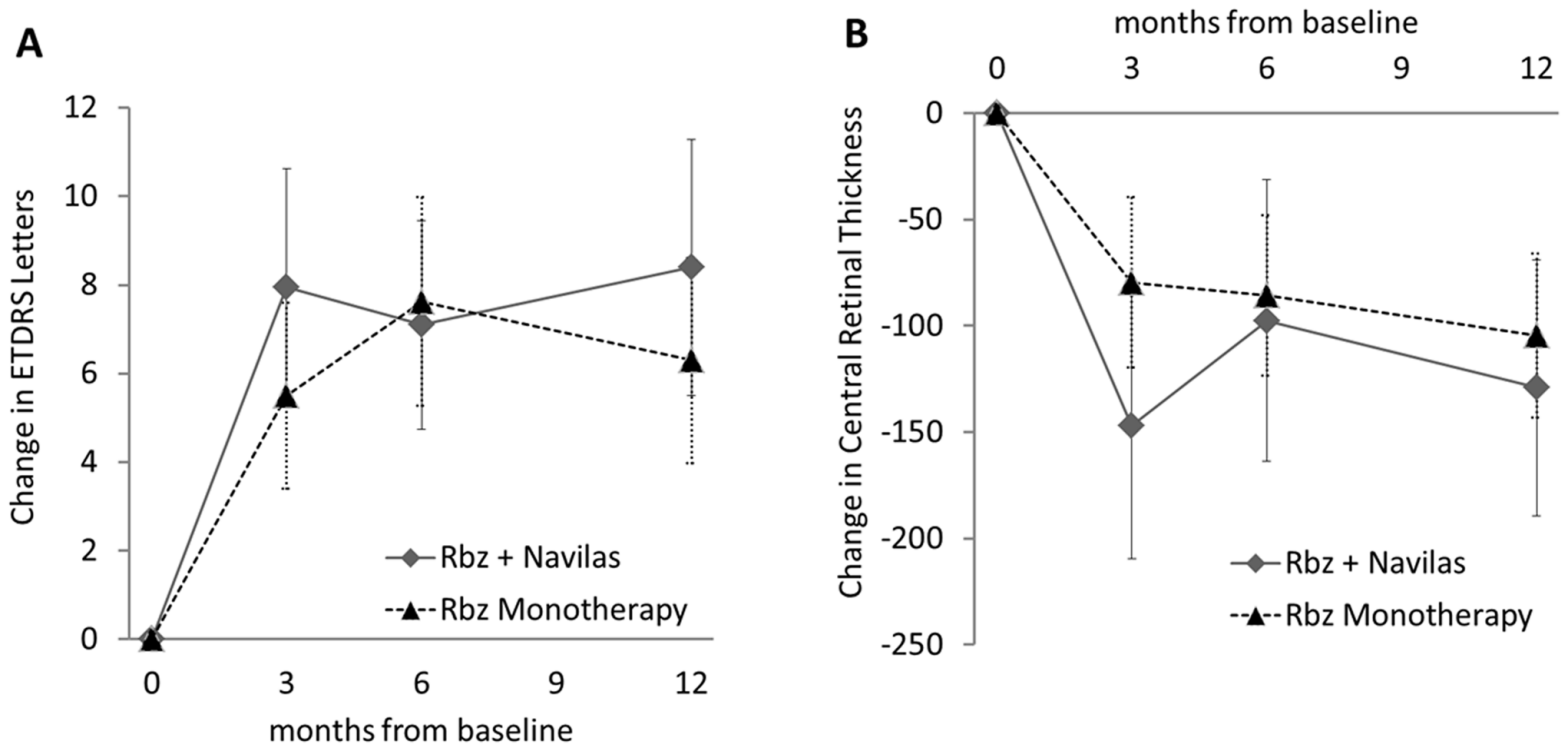
Course of best-corrected visual acuity (BCVA) and central retinal thickness (CRT). (A) Change in best-corrected visual acuity (BCVA) and (B) change in central retinal thickness (CRT) during 12 months follow-up (Error bars: 95% CI). Eyes received three Rbz loading injections and combination therapy eyes additionally received Navigated MLT 115±113 days mean from baseline. Thereafter, all eyes received PRN injections. Three months from baseline, combination therapy eyes had improved by a mean 7.9±7.6 letters and monotherapy eyes had improved by 5.5±5.8 letters (difference p = 0.150) and remained stable through the PRN phase. Twelve-month values were 8.4±8.3 letters and 6.3±6.5 letters, respectively (difference p = 0.258). Similarly, during 12 months CRT in the combination therapy cohort had improved by a mean -129±170 µm and in the monotherapy cohort from by a mean −105±107 µm (difference p = 0.487).

Immediately following the baseline exam, treatment was initiated with three ranibizumab intravitreal injections spaced approximately one month apart. By one month after this loading phase, both cohorts had reached equivalent and significant BCVA gains (Fig. 2, 3 months time point). Combination therapy eyes had improved by 7.9±7.6 letters and monotherapy eyes had improved by 5.5±5.8 letters (p = 0.150).

**Figure 3:**
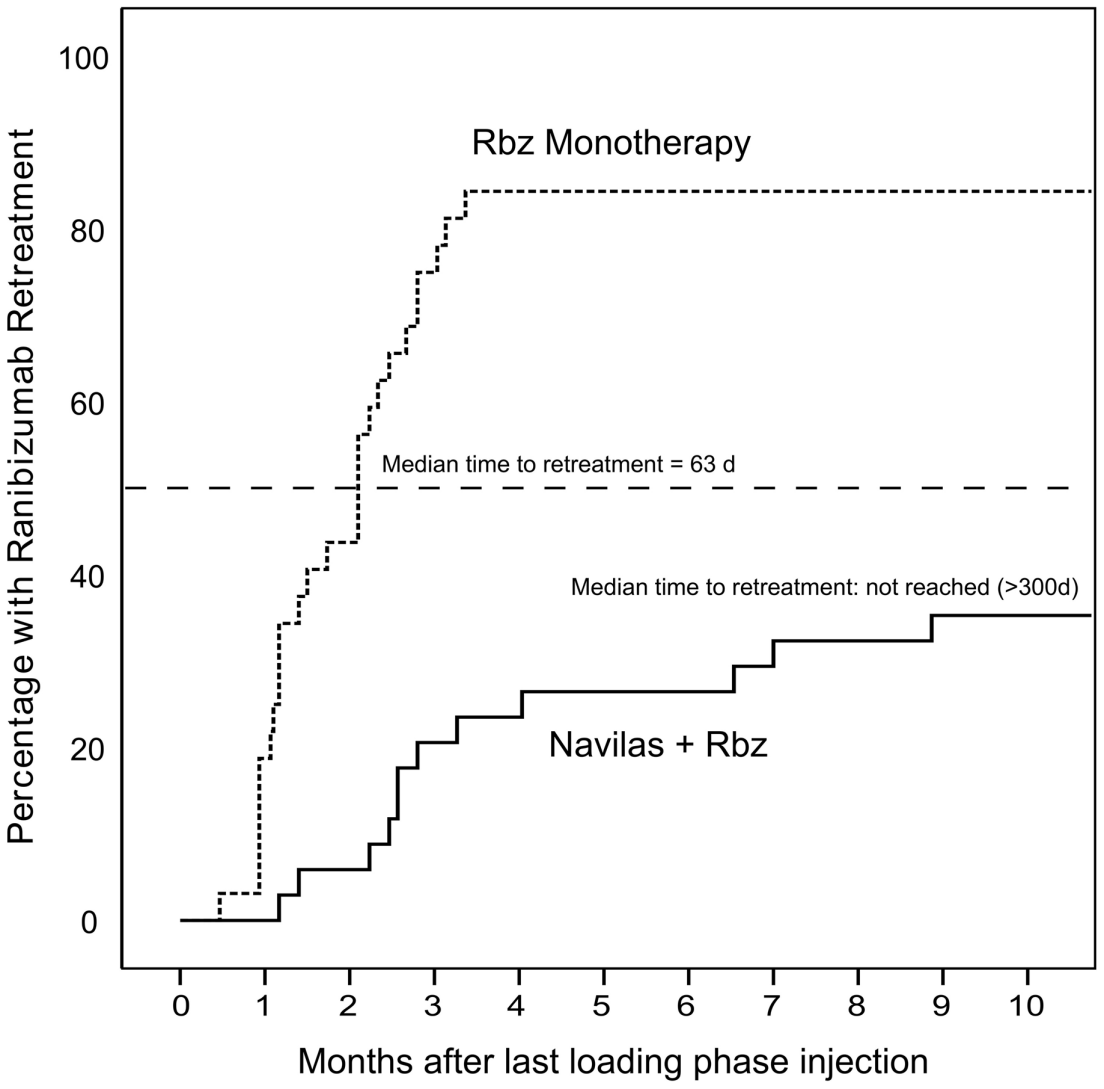
Kaplan-Meier analysis of injection retreatment after the last loading phase injection. A significantly higher proportion of Rbz monotherapy patients (84%) required injection retreatment compared to the navigated laser combination therapy cohort (35%, difference p≤0.001). Median time to retreatment was 63 and>300 days (median not reached during follow-up), respectively.

Navigated MLT was applied to combination therapy eyes 115±113 days mean after starting the ranibizumab intravitreal loading phase injections.

Initial BCVA gains remained stable in the PRN phase through months 6 and 12, with 6 month values of +7.1±6.2 letters and +7.6±6.5 letters in combination therapy and monotherapy eyes, respectively (p = 0.750).

By month 12 combination therapy eyes had improved 8.4±8.3 letters and monotherapy eyes had improved 6.3±6.5 letters. While there is a trend towards a better mean BCVA outcome for the combination therapy cohort, this difference did not reach statistical significance (p = 0.258).

Categorized BCVA outcomes are detailed in Table 2. By month 12, 47% of combination therapy eyes vs. 31% of monotherapy eyes had gained 10 letters or more and 21% vs. 9% had gained 15 letters or more. One patient in the combination therapy cohort had lost more than 15 letters and one patient in the monotherapy arm had lost more than 10 letters (3% of patients each).

**Table 2:** Best-Corrected Visual Acuity (BCVA) and Central Retinal Thickness (CRT) changes at month 12.

	Navilas + Ranibizumab n = 34	Ranibizumab Monotherapy n = 32	Test for difference (P-Value)
**BCVA change**			
− Mean ± SD	8.4±8.3	6.3±6.5	p = 0.258
− Median (range)	9 (−21 to +25)	6.5 (−12 to +17)	
−95% CI for mean	5.51, 11.31	3.99, 8.64	
**Categorized BCVA outcome (ETDRS letter score)**			
− Gain: 15 letters or more	21% (7)	9% (3)	p = 0.210
− Gain: 10 letters or more	47% (16)	31% (10)	p = 0.195
− Loss: 10 letters or more	3% (1)	3% (1)	p = 0.336
− Loss: 15 letters or more	3% (1)	0% (0)	p = 0.966
**Mean CRT ± SD (µm)**			
− Baseline	441±162	444±117	p = 0.928
−12 months	313±98	339±82	p = 0.255

SD, standard deviation; CI, confidence interval; CRT, central retinal thickness; BCVA, best corrected visual acuity; ETDRS, Early Treatment Diabetic Retinopathy Study.

Improvements in visual acuity were reflected by significant anatomic improvement from baseline: in the combination therapy arm central retinal thickness improved from 441±162 to 313±98 (mean improvement of −129±170 um) and in the monotherapy cohort from 444±117 to 339±82 (mean improvement of −105±107 um (Fig. 2); group difference p = 0.487).

### Retreatment rates and number of injections

Injection retreatment data in both cohorts was subjected to Kaplan-Meier analysis, which shows an early separation of the two curves (Fig. 3). The median time to retreatment calculated over all monotherapy eyes was 63 days, while at the end of the study at 10 months after the third injection (12 months of total follow-up) the median had not been reached in the combination therapy cohort. At the end of the study the combination therapy cohort had a significantly higher proportion of patients that required no further injections after the anti-VEGF loading phase than the monotherapy cohort: 65% vs. 16% (p≤0.001). The reduced retreatment rate in the combination therapy cohort corresponds to a significantly lower requirement for injections as compared to monotherapy: 0.9±1.2 vs. 3.9±2.3 after the loading phase, 3.9±1.3 vs. 6.9±2.3 total during 12 months follow-up, p≤0.001 for both comparisons. (Table 3)

**Table 3:** Analysis of required treatments.

	Navilas + Ranibizumab n = 34	Ranibizumab Monotherapy n = 32	Test for difference - P-Value
**Number of injections after loading phase (3x ranibizumab)**			
− Mean ± SD	0.9±1.2	3.9±2.3	p≤0.001
− Median (range)	0 (0 to 4)	4 (0 to 8)	
−95% CI for mean	0.45, 1.31	3.04, 4.71	
− Difference in mean number of injections vs. Ranibizumab Monotherapy (n, %)	−3.0, −77%		
**Total number of injections at 12 month including loading phase**			
− Mean ± SD	3.9±1.3	6.9±2.3	p≤0.001
− Median (range)	3 (3 to 7)	7 (3 to 11)	
−95% CI for mean	3.47; 4;35	6.04; 7.71	
− Difference in mean number of injections vs. Ranibizumab Monotherapy (n, %)	−3.0, −43%		
**Proportion of eyes with no need for injections after loading phase (%, n)**	65%, 22	16%, 5	p≤0.001
**Median time to retreatment (months)**	>10 (not reached during follow-up)	2.1	
**Number of navigated laser treatments Mean, Median (range)**	1.24, 1 (1–2)		
**Proportion of eyes with more than one navigated laser treatment**	24% (8)	N/A	

SD, standard deviation.

At 12 months from baseline, combination therapy eyes received a mean of 1.24±0.43 navigated laser treatments. Twenty-six eyes received the minimum of one navigated laser treatment and eight eyes received two treatments.

No adverse effects of intravitreal injections or navigated laser were observed during the study.

## Discussion

Diabetic macular edema is one of the most common reasons for significant visual impairment in the western world. It may affect people from all age groups and strongly affects patient's quality of life [9], [17].

The development of anti-VEGF therapeutics placed an important treatment option into the hands of the retinal physician to rapidly stabilize or even restore vision in DME. However, it is now apparent that chronic intravitreal injection therapy is needed to preserve or slightly extend these visual gains [4], [6].

This 12-month, prospective comparison on the efficacy of a standardized combination therapy regimen (three ranibizumab injections followed by navigated MLT) compared to anti-VEGF monotherapy, demonstrated that the combination of Rbz and navigated MLT may be superior in terms of retreatment rate and overall injection burden.

Both cohorts achieved significant and comparable visual gains attributable to the Rbz loading phase. Combination therapy gains were in trend higher (8.6 ETDRS letters) and non-inferior to Rbz monotherapy gains (6.3 letters, difference n.s.). Combination therapy patients had a significantly lower retreatment rate with 65% (vs. 16%) of patients not receiving further Rbz after loading/navigated MLT. On average, less than one injection was required after Rbz loading, corresponding to a reduction of 3 injections vs. Rbz monotherapy.(Table 3) Therefore, the application of navigated MLT after Rbz loading appears to reduce the injection burden considerably without compromising anti-VEGF visual gains.

With regard to similar combination therapies using conventional laser instead of navigated MLT, evidence from pivotal trials is inconclusive. In the RESTORE study, patients received a minimum of 3 initial injections until BCVA was stable at two consecutive visits and in one arm of this study patients received conventional laser photocoagulation at baseline and as needed at 3 month intervals [9]. As in this study, follow-up was 12 months from baseline, conventional laser combination therapy and Rbz monotherapy reached average BCVA gains of 6.1 and 5.9 ETDRS letters, requiring a mean of 7.0 and 6.8 injections, respectively. These insignificant differences between study arms indicate no benefit from adding conventional laser. Similarly, the DRCR.net trial using a different treatment and retreatment algorithm, did not demonstrate significant differences between a prompt laser arm and a deferred laser arm (no laser in 70% of patients during year one) [10]. Median visual gains were 9 letters each achieved with 8 and 9 injections median, respectively. In contrast, the smaller READ-2 study did show a reduced number of 4.9 vs. 9.3 injections in a 24-month period, when adding a mean number of 2.7 conventional laser treatments to anti-VEGF therapy. However, visual gains were slightly, but not significantly lower in the combination arm (6.8 vs. 7.7 letters gain) [18].

In summary, no consistent benefit from added conventional laser can be inferred. Reasons, besides a different study objective, may include the lack of standardization and accuracy of slit-lamp based laser application in the clinical setting.

Navigated MLT was developed to overcome these limitations with multimodal planning and treatment functions (“eye tracking”), e.g. accurately pinpointing microaneurysms on fluorescein angiography images and outlining edematous areas on OCT thickness maps for subsequent grid laser treatment. During treatment each spot is prepositioned and tracked with the physician remaining in control. We observed a significantly lower retreatment rate of navigated MLT monotherapy compared to conventional laser in a previous study, suggesting stabilization may be reached earlier after navigated therapy (i.e. frequently after the first treatment).

In contrast, most Rbz monotherapy patients in this study required retreatment within the first 3 months after Rbz loading (Median: 63 days). This may highlight the importance of using a standardized, fast-acting MLT that achieves most of its effects with the first treatment in order to reduce the requirements for additional injections.

Our study supports the results of a similar study conducted by the University of California in San Diego that included patients that had been treated with a similar standardized combination therapy regimen utilizing navigated MLT and bevacizumab. In these patients, an average of 4 injections were necessary during 12 months follow up period [19].

Generally, while these results suggest a compelling benefit of adding navigated MLT to anti-VEGF therapy, they await confirmation by larger multicentric, randomized controlled trials.

Considering the developments on drug based therapies together with the promising results of the presented study, we believe that the combination of two well-studied treatment modalities, intravitreal Rbz as well as navigated MLT offers the potential to improve DME management even further.
